# Platform Technology for Extended Reality Biofeedback Training Under Operant Conditioning for Functional Limb Weakness: Protocol for the Coproduction of an at-Home Solution (React2Home)

**DOI:** 10.2196/70620

**Published:** 2025-08-22

**Authors:** Anirban Dutta, Abhijit Das

**Affiliations:** 1 Centre for Systems Modelling and Quantitative Biomedicine University of Birmingham Birmingham United Kingdom; 2 Lancashire Teaching Hospitals NHS Foundation Trust Preston United Kingdom

**Keywords:** functional neurological disorder, functional movement disorder, extended reality, biofeedback training, experience-based co-design, EBCD, quality function deployment, QFD, rehabilitation, coproduction, artificial intelligence, AI

## Abstract

**Background:**

Functional neurological disorder (FND), including functional movement disorders (FMDs), arises from disruptions in the perception-action cycle, where maladaptive cognitive learning processes reduce the sense of agency and motor control. FND significantly impacts quality of life, with patients often experiencing physical disability and psychological distress. Extended reality (XR) technologies present a novel therapeutic opportunity by leveraging biofeedback training to target sensory attenuation and amplification mechanisms, aiming to restore motor function and the sense of agency.

**Objective:**

This study aims to coproduce and evaluate the usability of an XR technology platform for FND rehabilitation, focusing on functional limb weakness. The platform integrates biofeedback training with haptic and visual feedback to support motor relearning and control.

**Methods:**

We propose to use an experience-based co-design framework to engage patients with FND, caregivers, and health care professionals in collaboratively designing the XR platform. Stakeholders can share their experiences through narrative interviews and co-design workshops, which can identify emotional touchpoints and prioritized patient-centered needs. Insights will be synthesized through qualitative analysis and used to guide the development of system requirements via quality function deployment, ensuring that the platform aligns with user needs. XR training tasks—virtual reality relaxation, XR position feedback, and XR force feedback—will be integrated as needed into a unified therapeutic game experience through 4-week Agile sprints. Usability will be assessed using the System Usability Scale and qualitative feedback, with themes analyzed in NVivo to identify key areas for subsequent improvement.

**Results:**

High usability scores (>85) were recorded for the XR position feedback tasks in the predesign study, reflecting excellent usability and participant satisfaction. However, the virtual reality relaxation and XR force feedback tasks exhibited interindividual variability, underscoring the need for personalization. Key themes included customization, comfort, accessibility, and XR technological quality, ensuring that the XR platform effectively addressed diverse patient needs. The predesign study highlighted the potential of XR technology for FMD rehabilitation by integrating biofeedback training into a patient-centered game design framework. Approaches such as experience-based co-design and quality function deployment can support coproduction by systematically addressing usability and accessibility challenges. Brain-based metrics may further strengthen this evaluation. Accordingly, this study will use portable brain imaging to capture dynamic functional connectivity in key brain regions, enabling personalized interventions.

**Conclusions:**

Through coproduction and iterative refinement, this study aims to demonstrate the promise of personalized XR gaming technology as a scalable, at-home solution for FMD rehabilitation. In this context, personalization and accessibility are critical for optimizing usability and long-term clinical outcomes, paving the way for at-home implementation within the FND stepped care model.

**International Registered Report Identifier (IRRID):**

PRR1-10.2196/70620

## Introduction

### Background

Functional neurological disorder (FND), a condition in which individuals experience neurological symptoms (eg, weakness and tremor) without a structural brain lesion, is a complex disorder at the intersection of neurology and psychiatry, involving a dynamic interplay between brain-body processes and the mind [1]. A key explanation concerns a neurobiologically informed model of hierarchical Bayesian inference [2], which describes perception as a process of combining prior expectations and sensory input to minimize prediction errors [3]. FND symptoms arise when these prediction errors are not properly updated over time, leading to dysfunction in Bayesian perception and action [3]. Maladaptive, highly weighted prior expectations, influenced by bodily attention, are central to this process [2]. Empirical evidence supports this model, such as studies on altered predictive mechanisms in functional gait disorder [4]. In this context, functional movement disorders (FMDs), a subtype of FND [5], are atypical movement patterns (eg, tremor, dystonia, and weakness) not explained by organic disease, altered by distraction or nonphysiologic maneuvers and distinct from neurologic disease–related movement disorders. FMDs are common in neurology, accounting for 15% of new referrals, second only to headaches. FMDs have a poor prognosis, with 39% of patients remaining the same or worsening over time, often experiencing high physical disability and psychological issues [6]. These disorders present a clinical paradox: while the movements appear voluntary, patients experience them as involuntary and uncontrollable. The prevalence of FMDs among neurology patients in neurology clinics underscores the need for effective therapeutic interventions to address the significant disability associated with these disorders.

Recent research has highlighted the role of sensory attenuation and the sense of agency in the pathophysiology of FMDs [6]. Sensory attenuation refers to the brain’s ability to predict the sensory consequences of voluntary movement that suppress the actual sensory feedback, that is, reafference, a process that is crucial for distinguishing self-produced sensations from external stimuli [7]. In this predictive mechanism, a duplicate of the motor command, known as an efference copy, is postulated to be sent to a forward model that integrates the efference copy with an estimate of the body’s state to predict the sensory consequences of the movement. This predictive mechanism is postulated to be impaired in patients with FMD [8], reducing their ability to accurately predict the sensory consequences, thereby leading to the development of a reduced sense of agency over their self-generated movement [3]. This reduced sense of agency could explain why these patients perceive their movements as involuntary despite their voluntary appearance [9].

The sensory attenuation process is primarily mediated by forward models within the motor control system [7] that predict the sensory consequences of a planned action and modulate the reafference accordingly [10]. When an action is self-generated, the forward model predicts the resulting sensory input and attenuates the sensory experience to prevent self-caused sensations from interfering with the processing of external stimuli. Sensory attenuation can be measured using the force-matching task, where participants match a force applied to their finger either directly using their opposite hand (direct condition) or indirectly using a potentiometer and a torque motor (slider condition) [11]. Healthy individuals typically overestimate the force in the direct condition, producing greater matched force due to sensory attenuation. This phenomenon is reduced in individuals with FMD, potentially due to altered predictive mechanisms affecting their sensory attenuation process [8].

The force-matching task needs a well-designed haptic device, developed using robust methodological considerations [11], that can be combined with wearable brain imaging targeting key brain regions [3] for brain-behavior analysis. The primary motor cortex (M1) is involved in the execution of voluntary movements and plays a critical role in the sensory attenuation process [12]. Using the theta burst stimulation (TBS) approach, Voss et al [12] applied continuous TBS and intermittent TBS over the M1 to alter its excitability. The authors found that continuous TBS, which decreases cortical excitability, reduced sensory attenuation and improved performance in a force-matching task, suggesting a disruption in the predictive process. By contrast, intermittent TBS had no effect on sensory attenuation. The cerebellum also plays a crucial role in fine-tuning motor actions and predicting their sensory consequences [13]. The study by Blakemore et al [13] demonstrated that the cerebellum predicts the sensory outcomes of motor actions and signal discrepancies between expected and actual (afferent) sensory feedback. Positron emission tomography scans of participants performing a delayed tactile task showed increased cerebellar activity with longer delays, confirming its role in fine-tuning motor actions and motor learning by comparing intended and achieved movements. Within the cerebello-thalamo-cortical network, the thalamus acts as a subcortical relay station. Most afferents to the thalamus, whether ascending or cortical, are collateral branches of axons targeting lower motor centers, suggesting that thalamocortical pathways primarily monitor ongoing motor commands, with modulatory inputs adjusting information flow based on attentional demands [14]. The thalamus exhibits reduced activity during self-generated sensations, indicating its (relay) attenuation [15]. Hua et al [15] applied dynamic causal modeling analysis that indicated top-down modulation from the right inferior parietal lobe during sensory attenuation of an auditory stimulus. In this context, sensory attenuation is evident not only in somatosensory domains but also in auditory and visual modalities; for instance, self-generated sounds and visual stimuli are perceived as less intense than those generated externally, highlighting the broad applicability of sensory attenuation mechanisms across different sensory modalities. In FMDs, impaired sensory attenuation during movement may disrupt the cortico-cerebello-thalamo-cortical loop (a disruption referred to as dysrhythmia) [16], resulting in exaggerated or reduced movements, with both reflecting a reduced sense of agency over one’s own actions [17]. On the basis of a literature review of predictive coding and adaptive control in human-machine interfaces (HMIs; the systems or devices that enable interactions between users [patients] and robotic platforms or rehabilitation devices), Dutta [3] proposed the somato-cognitive action network (SCAN; a proposed brain network of intereffector regions within M1 that integrates motor planning with cognitive, physiological, and whole-body action control) as a key M1-based system linking effector and intereffector regions to support action intention, sensorimotor integration, and self-agency (or “awareness of being the agent of one’s own actions” [18]) by closing the cortico-cerebello-thalamo-cortical loop [16].

The 2 key theories of the sense of agency are the comparator model and the theory of apparent mental causation [19]. The right temporoparietal junction (rTPJ; a brain region implicated in the sense of agency and self-other distinction in movement control) is postulated to be a key node in the agency network for neuromodulation in FND [20]. However, the coordinates of the mean peak of activity in the temporoparietal junction (TPJ) have varied across studies [21], showing activation in the angular gyrus, middle temporal gyrus, parietal operculum, and supramarginal gyrus. This variation reflects the multidimensional processes at the TPJ contributing to the sense of agency, such as mismatch detection, action awareness, and sensory-motor conflicts. The angular gyrus is involved in intersensory mismatch detection, action awareness, and multisensory integration, while the supramarginal gyrus processes sensory-motor conflicts. Changes in perspective, mentalizing, and deception specifically activate the TPJ and posterior superior temporal sulcus, highlighting the role of perspective [22] in assigning motor actions to oneself or others. Overall, different neural mechanisms and specific brain areas within the TPJ contribute to the formation of agency when motor control is disrupted [3]. Functional neuroimaging has shown reduced activity and impaired connectivity of the rTPJ during functional tremor, indicating that altered rTPJ connectivity may play a contributing role. An impaired self-agency can be tested using intentional binding (motor intention is temporally matched with sensory feedback [23]) and sensory attenuation paradigms presented by Maurer et al [17]. The authors found decreased functional connectivity in patients with FMD between the rTPJ—crucial for self-agency—and bilateral sensorimotor regions, including the right sensorimotor cortex, bilateral cerebellum, bilateral supplementary motor area (SMA) [24], and right insula.

In contrast to sensory attenuation, sensory amplification occurs when the perceived intensity of a sensory stimulus is enhanced due to the influence of selective attention [25], for example, after error commission [26]. This effect is particularly noticeable when an individual directs their visual attention to a specific touch event, thereby increasing the perceived intensity of the touch. This phenomenon is related to the visual enhancement of the touch effect, where simply viewing (or even mentally imagining [27]) a body part can change tactile acuity for that part. Frontal and parietal cortices are involved in the modulation of sensory precision by attention that involves neural hierarchies [25]. Increased activity in the posterior parietal cortex is associated with enhanced sensory perception when visual attention is directed toward a stimulus, supporting the idea of optimizing sensory precision through attention. Empirical studies have shown that visual attention to a touch event, whether active or passive, amplifies the perceived intensity of the touch. This sensory amplification effect is significantly larger than the sensory attenuation effect, underscoring the powerful influence of attention on sensory perception. Overall, the interplay between sensory attenuation and amplification highlights the complex nature of sensory processing, where the brain dynamically adjusts the perceived intensity of stimuli based on predictive models and attentional focus [10,25]. Understanding these sensory mechanisms provides valuable insight into how sensory experiences are modulated in various contexts, including during the observation of others’ actions and in conditions involving altered sensory processing, such as FMD [8]. In understanding sensory mechanisms, it is important to distinguish between interoception, the sense of internal bodily states, and exteroception, which relates to the perception of the external environment through senses. Pain, for instance, can be interoceptive when it arises from within the body; and, in some cases, dysfunctional sensory amplification due to the overweighting of nociceptive input can lead to exaggerated prediction errors, reinforcing and intensifying the perception of pain [28], a common feature in FND-related pain symptoms and diagnoses [29].

We aim to leverage the interplay between sensory attenuation and amplification in exteroception, which is crucial for action in the external environment, by applying an operant conditioning–based implicit learning approach [30]. While this method can also be applied to interoception, this study focuses specifically on external action. This approach has shown therapeutic potential in addressing learned nonuse, a condition thought to arise from a maladapted forward model, in individuals with chronic stroke [31]. Our operant conditioning technique reinforces desired motor behaviors using visual feedback by dynamically adjusting gain and noise, with the goal of reweighting sensory attenuation and amplification [30]. This multilevel stimulation process adjusts prediction errors by aligning predicted and actual sensory input within the brain’s predictive coding framework [30]. Kumar et al [31] showed the feasibility of the virtual reality (VR)–based balance training system that used the operant conditioning approach proposed by Dutta et al [30], where higher weighting of the center of pressure from the paretic limb for visuomotor control implicitly encouraged patients with hemiplegic stroke to engage the affected limb during weight-shifting gaming tasks. Intermediate catch trials provided unbiased measures of standing balance capability. This implicit learning approach incorporated individualized weight distribution for VR tasks as well as subtle reinforcement (including suggestions) during VR gaming as an operant factor [32] that improved paretic limb behavior.

### Hypothesis for Operant Conditioning Sense of Agency

**Figure 1:**
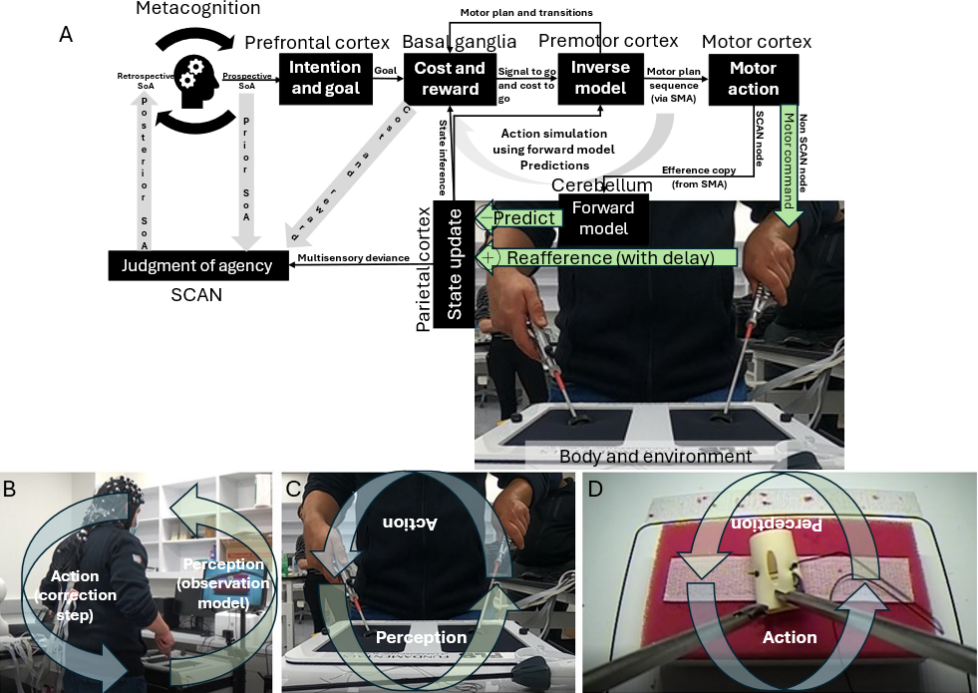
Neurocomputational framework of the sense of agency (SoA) and motor control during the action-perception cycle from perception of error (observation model) to the corrective action (correction step). (A) Schematic of neural mechanisms underlying SoA and motor control, illustrating cortical and subcortical interactions across prefrontal cortex, basal ganglia, premotor cortex, motor cortex, cerebellum, and parietal cortex. The supplementary motor area (SMA), involved in internally generated motor planning, is functionally integrated within the somato-cognitive action network (SCAN). The proposed framework integrates intention and goal setting, cost-reward evaluation, internal forward and inverse models, sensory prediction, and reafference processing. Multisensory deviance contributes to SoA judgments via the SCAN. (B) Illustration of action correction step during laparoscopic simulation training using a fundamental of laparoscopic surgery (FLS) trainer box, highlighting the correction loop in response to perceptual error observed using camera feedback on a video monitor. (C) Action-perception loop during active tool manipulation in the FLS trainer box environment, emphasizing real-time integration of motor commands and perceptual feedback. (D) Camera close-up of task performance using laparoscopic tools for perception-action cycle, illustrating the observation model and its role in modulating corrective actions.

**Figure 2:**
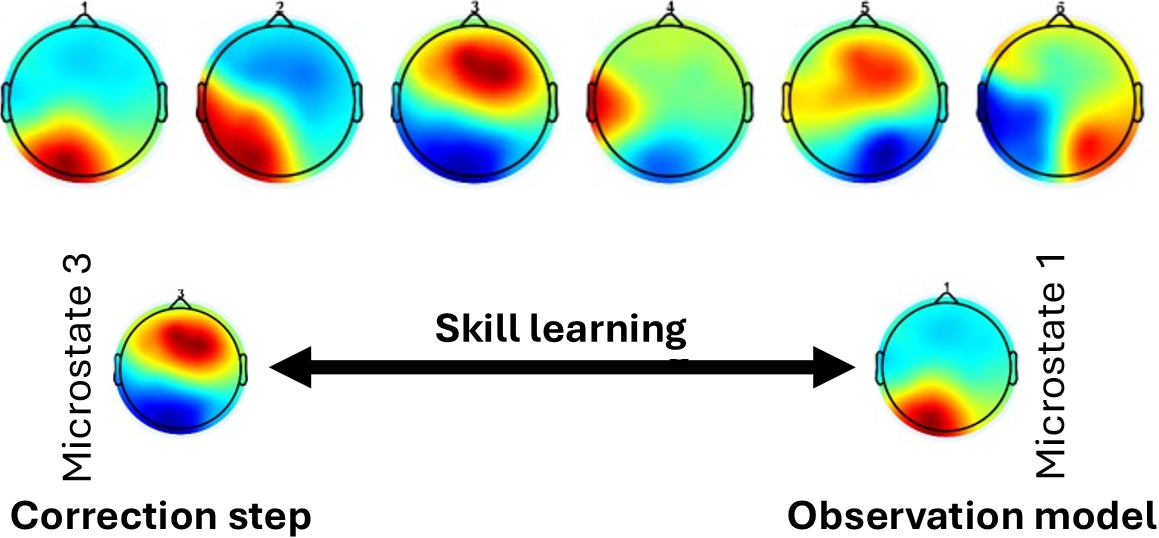
Interpretation of microstate transitions in expert and novice groups within the Bayesian predictive coding framework (adapted from Walia et al [33], which is published under Creative Commons Attribution 4.0 International License [33]). During learning, the brain alternates between acting as an observer and as a controller: the “brain as controller” model (correction step) depends on predictions generated by the “brain as observer” (observation model), and vice versa. This dynamic is particularly relevant to switching state-space models in neuroimaging.

Figure 1 shows the neurocomputational framework of agency and motor control [3] illustrated for fundamentals of laparoscopic surgery (FLS) training with a camera-equipped FLS trainer box [33]. It depicts how voluntary movement and the sense of agency are generated through a brain-body loop involving a prospective sense of agency leading to intention, prediction, and feedback. The prefrontal cortex (PFC) sets goals based on a prospective sense of agency, which are evaluated by the basal ganglia for cost and reward before being translated into motor commands via inverse and forward models. The cerebellum predicts the sensory consequences of action, and delayed reafferent signals from the body (eg, touch and position) are compared against these predictions. Discrepancies (prediction errors) update the internal state of the parietal cortex and feed into the SCAN for retrospective agency judgment. We found that portable brain imaging can capture error-related brain-behavior relationships during complex motor tasks [33], as demonstrated in Figure 2, by revealing distinct microstate transitions between expert and novice groups within the Bayesian predictive coding framework. Together, the prospective and retrospective senses of agency support metacognition and are crucial for rehabilitation using extended reality (XR; an umbrella term encompassing VR, augmented reality, and mixed reality technologies), where reestablishing predictive control and agency is key, particularly in disorders such as FMD.

**Figure 3:**
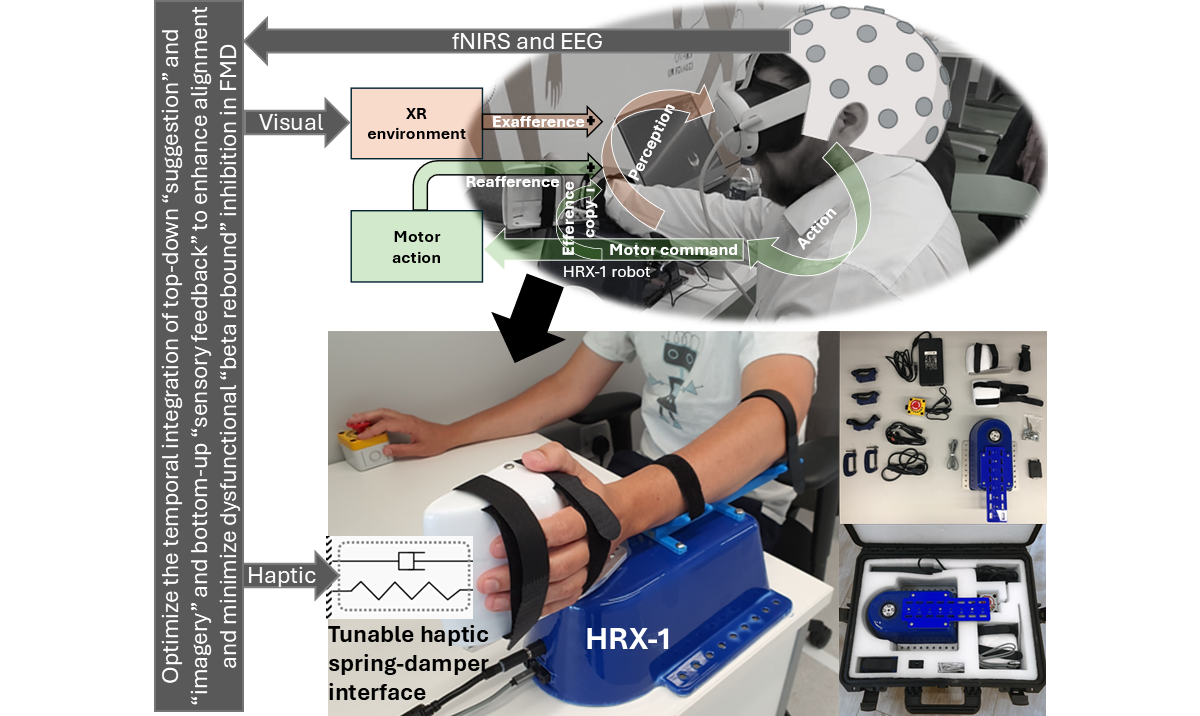
The modular HRX-1 is a haptic robotic device that integrates functional near-infrared spectroscopy (fNIRS), electroencephalography (EEG), and machine learning to support both top-down and bottom-up exafferent modulation within an extended reality (XR) environment. Its aim is to reduce abnormal regulation of EEG beta-frequency activity with EEG-fNIRS joint-imaging facilitating its localization in the supplementary motor area, which is commonly observed in Functional Movement Disorder (FMD). In FMD, patients often lack the typical beta desynchronization that occurs before and during movement. This suggests their brains may struggle to effectively "release the brake," transitioning from motor planning to execution, which contributes to symptoms such as tremor, weakness, and abnormal movements.

The central hypothesis is that a prospective sense of agency, which enables self-initiated movement intentions, can be generated in a top-down manner through “suggestion and imagery” within an XR environment. This process can be monitored using slow movement-related cortical potentials (MRCPs) [34], such as the early readiness potential originating from the SMA complex (Bereitschaftspotential I) to the SCAN regions of the M1 (Bereitschaftspotential II). At the intereffector SCAN regions, MRCP is postulated to begin with a slow negative shift around 1.5-2 seconds before movement onset, peaking negatively at movement onset, which coincides with a sharp increase in event-related desynchronization in the mu and beta bands. While event-related desynchronization is reflected in non-SCAN regions of M1, the intereffector SCAN regions are postulated to support body integrated action planning. Within the XR context, exafferent sensory input will be modulated following self-initiated motor execution to integrate with reafferent feedback through postulated Bayesian fusion [3], thereby influencing prediction error dynamics that arise from a disrupted forward model, as observed in FMD [35]. Following movement execution (postmovement positivity in MRCP), beta event-related synchronization signals the return to a baseline state. In this context, through operant conditioning embedded in XR-based gaming rewards, implicit learning is postulated to drive the recalibration of the forward model, progressively reducing reliance on exafferent input and supporting the restoration of normal motor function (Figure 3).

Voluntary movement is initiated by neural activity that first appears in the rostral cingulate zone within the dorsal anterior cingulate cortex, followed by activation in the pre-SMA and SMA regions and then in the M1 [36]. All these regions send projections to the spinal cord, with the M1 serving as the primary conduit for transmitting motor commands via the corticospinal tract. Concurrently, an efference copy of the motor commands is sent to the primary somatosensory cortex, cerebellum, and striatum, where it supports real-time correction, motor learning, and the inhibition of competing movements. We propose that during the preparatory stage of a voluntary motor task, efferent signaling from the SMA complex [24] sets the initial conditions by modulating the SCAN node within the M1 [3], thereby priming the non-SCAN effector regions for context-appropriate action. We suggest that this contextual modulation of the SCAN node by the SMA is impaired in FMD [24,37], which can lead to persistent fatigue [38]. Moreover, stress-related mechanisms in FMD may arise from overlapping adrenal connectivity and medial motor regions (the SMA and dorsal anterior cingulate cortex) disrupting the integration of motor intention and bodily arousal [36].

Building on the findings of Maurer et al [17] that link impaired self-agency to disrupted connectivity between the rTPJ and sensorimotor regions, we propose using simultaneous mobile brain and body imaging (brain-behavior joint monitoring [33,39]) to monitor intentional binding and efferent signaling. In this context, action-perception coupling monitoring [39] will leverage task-specific dynamic functional connectivity (dFC; a method for assessing time-varying brain network interactions during tasks) [40] between the rTPJ and sensorimotor cortex to assess abnormalities in sensory feedback processing in FMD, specifically, dFC between the rTPJ and the SMA complex [24], which contributes to efferent signaling and intentional binding [41-43] from the pretask to posttask phases, respectively, in the action-perception cycle.

In FMD, increased (localized to the left SMA [37]) and persistent beta synchronization [44] disrupts motor preparation, diverging from the typical role of brief beta bursts in facilitating cognitive control and goal-directed behavior [45], that is, “brain as controller.” We propose that within the sensorimotor cortex, mu rhythms support the brain’s role as an “observer” [22] engaged in action observation, salience detection [46], and contextual gain setting [47], while beta rhythms reflect the brain’s role as a “controller,” governing motor execution and precision [48]. In this context, it is further postulated that mu rhythms are linked to SCAN-like intereffector zones and the cingulo-opercular (salience) network [36], whereas beta rhythms are more specifically tied to non-SCAN effector-centric motor control in the M1. These sensorimotor rhythms alternate in bursts to modulate corticospinal excitability in healthy individuals, that is, motor-evoked potential amplitudes measuring corticospinal excitability peak during the beta rhythm’s peak-to-falling phase and the mu rhythm’s trough-to-rising phase in the sensorimotor cortex [49]. The beta rhythms extend to broader motor-related regions, including the basal ganglia, and play a role in motor control and movement stabilization crucial in “brain as controller.” The dynamic interplay of mu and beta event-related desynchronization and event-related synchronization, which is essential for normal motor function, is disrupted in FMD and may be restored through electroencephalography (EEG; a technique for recording electrical activity of the brain, used here for assessing cognitive and motor states) via biofeedback-driven training, which sequences the execution of an observation model through to the correction step [35,50] in XR-based motor tasks (Figure 1).

The amygdala has functional and anatomical connections to cingulo-opercular network nodes such as the anterior insula and anterior cingulate cortex [51]. FMD is postulated to involve maladaptive cognitive learning from aversive stimuli, leading to overactivity in the amygdala [52]. A heightened fronto-amygdala connectivity can disrupt the left PFC’s role in flexible, top-down cognitive control [53]. Therefore, our operant conditioning approach in XR begins with a relaxation and deep-breathing activity [35] combined with vagus nerve stimulation [54]. This is followed by a motor relearning task using a haptic device, rooted in the behaviorist framework formulated by Skinner [55] and formalized through reinforcement learning [30], designed to deliver implicit rewards and promote the adaptation of the internal forward model through gaming behavior [35]. This process relies heavily on the cerebellum for prediction error coding to facilitate effective motor adaptation to exafferent signaling for reinforcement learning [56]. In this context, portable neuroimaging also enables the investigation of individual activity in the brain regions such as the ventrolateral PFC, superior parietal lobule, supramarginal gyrus, angular gyrus, dorsolateral PFC, frontal eye field, and premotor cortex, and M1 [33,39]. Within the frontoparietal network, a distinction is postulated between attention driven by unexpected stimuli (bottom up) and attention that is controlled and goal directed (top down) [33]. The ventral frontoparietal network, including regions such as the temporoparietal cortex and inferior frontal cortex, is specialized for detecting behaviorally relevant stimuli, particularly when they are salient or unexpected (bottom up). This network acts as a “circuit breaker” for the dorsal system, directing attention to salient events. By contrast, the dorsal frontoparietal network, comprising areas such as the intraparietal sulcus and frontal eye fields, is primarily involved in goal-directed, top-down attentional control. This system is engaged during focused attention on specific targets or tasks. Understanding the interplay between these brain networks using switching state-space models for neuroimaging [57] is crucial during operant conditioning in FMD. In this context, the information flow model for developing the sense of agency [58] involves a leading network for mismatch detection, including the right supramarginal gyrus, left anterior inferior parietal lobule, anterior insula, and rTPJ. Mismatch information is relayed through intermediate areas before reaching the lagging network, which includes the bilateral PFC, cingulate cortex, and bilateral posterior inferior parietal lobule. The role of the PFC in the lagging network suggests its later involvement in retrospective sense of agency processing.

### Related Prior Works

Expertise in the FLS “suturing and intracorporeal knot-tying” task was assessed through error-related brain states using portable, multimodal brain imaging [33]. The study by Walia et al [33] revealed significant differences in EEG microstate dynamics between experts and novices during complex surgical task training, aligning with the microstate taxonomy formulated by Bréchet et al [59]. Microstate 1 (comparable to microstate C [ventral visuospatial attention] in the taxonomy formulated by Bréchet et al [59]) and microstate 3 (comparable to microstate D [focus switching and reorientation]) were dominant across conditions. Experts lacked microstate 2 (comparable to microstate A [exploration processes]) during error epochs, indicating reduced exploratory motor responses and a more efficient exploitative strategy. Experts transitioned frequently to microstate 3 for error correction, while novices relied heavily on microstate 1 and transitioned back and forth between microstates 1 and 3, reflecting ongoing attempts at skill learning (Figure 2). In addition, experts exhibited prefrontal activity (microstates 5 and 6) preceding transitions to microstate 3, while novices showed left-right transitions (microstate 4) to microstate 1. Thus, microstate analysis highlighted that experts use refined attentional dynamics and reduced exploratory activity during errors, consistent with the neurophysiological underpinning described by Bréchet et al [59]. Recently, we used wearable functional near-infrared spectroscopy (fNIRS; a noninvasive brain imaging method using light to measure changes in cerebral blood oxygenation) [60] to assess brain activation during simulated operative dictation in an ecologized enactive framework. The operative dictation of a surgical task with “brain as controller” relies on first-person mental imagery—“brain as observer” [22]—to consciously recreate and manipulate past action experiences as mental representations without physical stimuli (refer to the “action simulation” loop in Figure 1). Simultaneous expert feedback and cues (exafferent external suggestions) can enhance enactment imagination, facilitating beneficial updates to the internal models.

The cerebellum plays a crucial role in reshaping the relationship between motor intention, action, and outcome through its connectivity with the medial PFC, as well as its interactions within the thalamocortical circuitry, including the cerebellum-thalamus-SMA pathway, for sensory gating mechanisms in the correction step (Figure 2) [61]. This dynamic interaction in the “action simulation” loop (Figure 1) likely provides a foundation for hypnosis to modulate the sense of agency [62]. Evidence suggests that the cerebellum contributes to the prediction and coordination of motor actions, integrating sensory feedback to refine the alignment between motor intentions and perceived outcomes [13]. Hypnosis, through its effects on attention and sensory modulation, may influence these cerebellar processes, which can be facilitated with noninvasive brain stimulation [63,64], enhancing the precision of prediction errors [56,65]. The study by Polito et al [66] highlights how hypnotic suggestions can disrupt habitual motor-sensory loops, potentially recalibrating the perception of control over actions. In addition, Blakemore et al [62] demonstrated that hypnosis can modify the temporal binding of intentional actions and their outcomes, further supporting its capacity to reshape the sense of agency through altered cerebellar and SMA connectivity. These insights suggest that hypnosis, by interacting with the neural substrates involved in sensory gating and motor planning, has the potential to refine the brain’s representation of agency, particularly in conditions where the sense of agency is disrupted.

A recent study by Bréchet et al [67] examined how intense meditation training modulates EEG microstates, revealing significant postmeditation topographical changes. Two novel microstate topographies emerged after meditation, explaining nearly 50% of the data variance, and were localized to brain areas involved in self-related multisensory experiences, including the insula, supramarginal gyrus, and superior frontal gyrus. Unlike prior studies that observed stability in microstate topographies [68], meditation induced notable deviations from the premeditation and placebo conditions, including the absence of microstate C (postulated in “observation model” in Figure 2) dominance after meditation. These findings align with meditation-induced changes in consciousness, such as enhanced self-observation activity, which may increase during meditation-primed self-hypnosis [22]. The study by Katayama et al [69] explored EEG microstates during different stages of hypnosis, ranging from resting, light hypnosis, deep hypnosis, and recovery, to understand changes in brain activity associated with altered states of consciousness. The “classic” 4 classes (A, B, C, and D) were identified, with microstates A and C increasing in duration, occurrence, and time coverage during deep hypnosis, while microstates B and D decreased. Resting and recovery conditions exhibited intermediate microstate characteristics between light and deep hypnosis, indicating distinct trajectories in the transition to hypnotic states. The findings suggest that deep hypnosis prioritizes specific cognitive processes related to the postulated “brain as observer,” enhancing insight [22] while reducing executive processes related to the postulated “brain as controller.” Comparisons with other states revealed similarities between deep hypnosis and schizophrenia (eg, reduced executive control) and between light hypnosis and meditation (eg, relaxation and heightened awareness). Overall, the study by Katayama et al [69] demonstrates that EEG microstates systematically evolve with hypnotic depth, reflecting shifts in the brain’s cognitive and functional dynamics. In this context, the postulated “brain as observer” approach [22] highlights the functional contributions of the anterior and posterior subsystems of the default mode network (DMN) and their connectivity during future-oriented thought; for example, combining insight meditation (with self-hypnotic induction) to support “brain as observer” and enactment imagination (with suggestions) to enhance “brain as controller” may help modulate FMD symptoms. In the related study by Xu et al [70], participants reported higher levels of vividness and self-projection when reflecting on their future self, highlighting the dominant cognitive function of future-self imagery. A functional dissociation was observed within the DMN: the anterior DMN was more active during present-self reflection, associated with self-referential processing (“brain as observer”), while the posterior DMN was preferentially activated during future-self reflection, linked to mental scene construction (“brain as controller”). These findings highlight the distinct roles of DMN subsystems in future-self oriented thinking to escape the present brain-body FND state, offering insight into the neural basis of the “brain as observer” versus the “brain as controller” functions [22].

### Goal of This Study

“Suggestion” and “imagery” are key tools in psychotherapy. For operant conditioning in XR [35], mental imagery can help patients reimagine movement scenarios with positive outcomes. Suggestions (with or without hypnotic induction [71]) can be used to challenge distorted beliefs about limb weakness with evidence [72] and promote adaptive perspectives. Priming new perspectives [73] can be strengthened with positive sensory feedback using an adaptive XR-based HMI to simulate “normal” movement state transitions [74]. This study introduces an innovative HMI approach by combining top-down and bottom-up attention facilitation to develop a sense of agency in an engaging XR gaming environment; for example, participants may lack task-error perception in the lagging network [58], showing low medial frontal cortex activity. In this context, portable brain imaging of medial frontal cortex activity can guide participant-specific motor control interventions to improve action-perception coupling via intentional binding using a haptic robotic device (HRX-1; a modular haptic robotic device developed by HumanRobotiX Ltd, used in this study for force feedback and biofeedback-based motor training; Figure 3). Our approach also aims to uncover—using brain-behavior monitoring—how the sense of agency develops through action-effect contingencies using tunable haptic feedback and XR force-matching tasks [3,11] under the action-effect binding paradigm [75].

In this study, we examine perception-action binding in FMD within the predictive coding framework [3], focusing on dFC across key brain regions, including the sensorimotor cortex, the pre-SMA and SMA regions (SMA complex [24]), and the rTPJ, all of which are essential for motor preparation, control, and the sense of agency [43,76]. In stimulus-cued movement, postmovement beta synchronization both predicts behavioral stimulus–response binding and is significantly elevated in patients with FMD compared to healthy controls [37]. Midcentral beta rebound reflects the electrophysiological “resetting” of overlapping networks in the motor area and SMA [24], also linked to visual cued motor imagery [77]. Persistent beta synchronization during motor preparation, not just after movement, may reflect abnormal explicit control of movement in FMD [44]. This likely results from excessive attentional focus on the act of moving itself, with attention misdirected away from the movement’s goal and instead fixated on the mechanics of movement. This suggests active monitoring of the state [3] (Figure 1), which is likely driven by the perceived risk and uncertainty within the intermittent control framework [78].

In FMD, the normal intermittency of active feedback control characterized by on-off switching seems to be replaced with active monitoring of the state [3], potentially due to perceived risk and uncertainty reflected in persistent beta synchronization [78]. While directly correlating on-off switching with beta desynchronization-synchronization is complex in real-world settings, this study aims to explore this relationship in a controlled XR environment where stochastic movements may reveal meaningful patterns. In addition, perceived risk and uncertainty can be modulated in an XR environment while probing intermittent control in individuals with FMD versus healthy controls. In this context, we suggest that “action simulation” as active observer originating from SCAN nodes within the M1 (Figure 1) operates in parallel with the motor execution loop, which arises from non-SCAN effector-specific regions in the M1 responsible for motor commands (postmovement beta rebound is generated in the motor cortex [79]). During motor imagery, this execution pathway is inhibited, allowing “action simulation” to dominate. Predictions generated by the “action simulation” active observer are integrated with reafference from body sensors via Bayesian fusion that may not always be optimal [3]; for example, in individuals with FMD, impaired beta synchronization may reflect dysfunction in this fusion process, potentially resulting in the disproportionate weighting of internally generated active observer predictions from the action simulation loop over actual sensory evidence from body sensors. We propose to normalize this fusion process through the integration of top-down motor imagery (“action simulation”) and bottom-up sensory feedback from body sensors modulated with exafferent inputs in the XR gaming environment. Our co-designed XR-based HMI aims to offer an artificial intelligence (AI)—and machine learning (ML)—enabled personalized platform for delivering a game-based intervention for home rehabilitation [35].

Prior studies on intentional binding [76,80,81] suggest that repeated operant experience can strengthen associations, linking it to lower-level, implicit aspects of the sense of agency through automatic associative learning. Prior studies also highlight a distinction between implicit and explicit aspects of the sense of agency. In this context, the conscious expectancy in conscious, reflective judgment of the sense of agency can be developed by showing patients with functional motor symptoms their physical signs, such as the Hoover sign or tremor entrainment [82]. Automatic link formation with associative learning for a prereflective, automatic experience of the sense of agency can be developed via repeated operant experiences as shown in our learned nonuse cases [31]. While the implicit and explicit aspects of the sense of agency are separable, they interact through the same top-down and bottom-up mechanisms [81] that can be artificially modulated in the XR through exafference (Figure 3). Therefore, XR with exafference holds significant potential for advancing the understanding, diagnosis, and treatment of FMD. The recent review by Brouwer et al [74] highlighted how VR can be used to explore neurocognitive mechanisms, such as the sense of agency, attention, and suggestibility, and proposed VR-based interventions that could offer novel therapeutic avenues. The premise is that VR can manipulate and study predictive coding abnormalities to uncover the mechanisms of FMD where the key concepts include attention, the sense of agency, and suggestibility.

A compelling recent study [83] showed that multidisciplinary treatment comprising physiotherapy and psychotherapy can significantly improve the physical component of quality-of-life outcomes in patients with FMD. However, a lack of specialists means that access to these treatments is sparse. Even when available, the cost for patients to attend regular hospital sessions can be financially prohibitive. Lord Darzi’s 2024 report further highlighted the need to bring care closer to home by aligning financial incentives and prioritizing technology [84]. Our proposal is therefore ideally timed [85], aiming to bring cost-effective and accessible VR rehabilitation [74] into the homes of people with FMD. We aim to create a scalable, home-based rehabilitation solution that shifts care from clinic to home with a stepped care model [86,87]. Home care reduces the need for travel; promotes sustainable health care; and offers accessible, personalized rehabilitation. Our approach addresses the growing demand for remote health care and will provide equitable and cost-effective therapies. The proposal also includes a product delivery road map with technoeconomic analyses applied to the stepped care model. This stepped care model proposed for Neurorehabilitation Online [88] can lower health care costs, encourage adoption, and expand the solution’s use to other rehabilitation needs.

## Methods

### Ethical Considerations

The predesign study, part of patient engagement and technology coproduction, was classified as service development and quality improvement, not requiring full National Health Service (NHS) ethics review. All participants gave written informed consent, and the study followed the Declaration of Helsinki principles. For this study, ethics approval will be obtained from the relevant institutional review boards and the health research ethics committee at Lancashire Teaching Hospitals NHS Foundation Trust before commencing each research stage. All participants will provide written informed consent before participation. They will receive accessible materials explaining the project’s goals, methods, and potential risks, emphasizing their right to withdraw at any time. All personal and biometric data will be anonymized and encrypted. Data management protocols adhering to ISO (International Organization for Standardization) 27001 standards will be implemented to ensure confidentiality and secure storage, in compliance with the General Data Protection Regulation. Participants involved in narrative interviews will review and approve video content intended for research dissemination. They retain the right to withdraw their consent for the use of their video at any stage.

### Participant Population

This study will recruit adults aged ≥18 years with a confirmed diagnosis of functional limb weakness, a well-characterized subtype of FMD. Functional limb weakness is among the most common presentations of FMD and is particularly suited for XR-based motor retraining due to the clarity of its diagnostic criteria (eg, the Hoover sign [72]), the reproducibility of motor tasks in VR environments [35], and alignment with clear rehabilitative goals [89]. This focus aligns with emerging evidence supporting the feasibility, usability, and potential of hypnotherapy in this subgroup [90]. All participants will be assessed by a consultant neurologist using positive diagnostic signs consistent with *DSM-5* (*Diagnostic and Statistical Manual of Mental Disorders* [Fifth Edition]) criteria and established clinical features (eg, the Hoover sign and variability with distraction). This study will emphasize safety, usability, and the personalization of XR-based interventions in the home setting.

### Inclusion and Exclusion Criteria

The inclusion and exclusion criteria are presented in Textbox 1.

**Inclusion and exclusion criteria.**

**Inclusion criteria**

- Adults (aged ≥18 y)
- Confirmed diagnosis of functional limb weakness by a neurologist using positive clinical signs
- Able to understand and provide informed consent
- Willing and able to participate in home-based extended reality (XR) interventions
- Access to a home environment suitable for safe XR device use

**Exclusion criteria**

- Presence of co-occurring neurological disorders with structural pathology (eg, stroke and multiple sclerosis)
- Other subtypes of functional movement disorder (eg, tremor and dystonia)
- Severe cognitive impairment or psychiatric comorbidity interfering with participation
- Inability to safely use XR equipment at home

### Recruitment Strategy

Participants will be recruited through neurology outpatient clinics and the specialized FND service affiliated with the Royal Preston Hospital in Preston, Lancashire, England. Treating clinicians will identify eligible individuals during routine care, and interested participants will be referred to the study team for consent and screening. Partnerships with FND charities and patient advocacy groups will support broader outreach and ensure inclusive recruitment.

### Experience-Based Co-Design

The research domain criteria (RDoC) framework [91], developed by the National Institute of Mental Health, offers a dimensional approach to understanding shared pathways, which can guide the development of targeted neurotechnological innovations. Unlike traditional diagnostic methods that focus on symptom-based categories, the RDoC framework seeks to understand disorders by integrating multiple levels of information, including neuroscience and behavioral science. The adoption of a dimensional, neuroscience-based framework is proposed to facilitate better phenotyping of heterogeneity and the identification of biomarkers, potentially leading to more precise and effective rehabilitation technologies [92].

While the RDoC framework offers a comprehensive approach to explaining the diagnosis and developing more personalized rehabilitation technologies, Spagnolo et al [92] acknowledge practical challenges, particularly the complexity of integrating multiple levels of analysis into the clinical care pathways. To address this, we propose using experience-based co-design (EBCD; a participatory method involving patients, caregivers, and clinicians in designing health care solutions based on lived experience) [93] adapted for technology integration with the proposed optimum clinical pathway for adults with FND [94]. Rehabilitation technologies encompassing HMIs that access, monitor, investigate, assess, manipulate, and emulate neural systems present significant ethical concerns due to their profound impact on human identity, autonomy, privacy, and well-being. In this context, engaging patients and stakeholders is essential for conducting health care economic analyses and addressing ethical issues in implementing rehabilitation technologies for FND.

**Figure 4:**
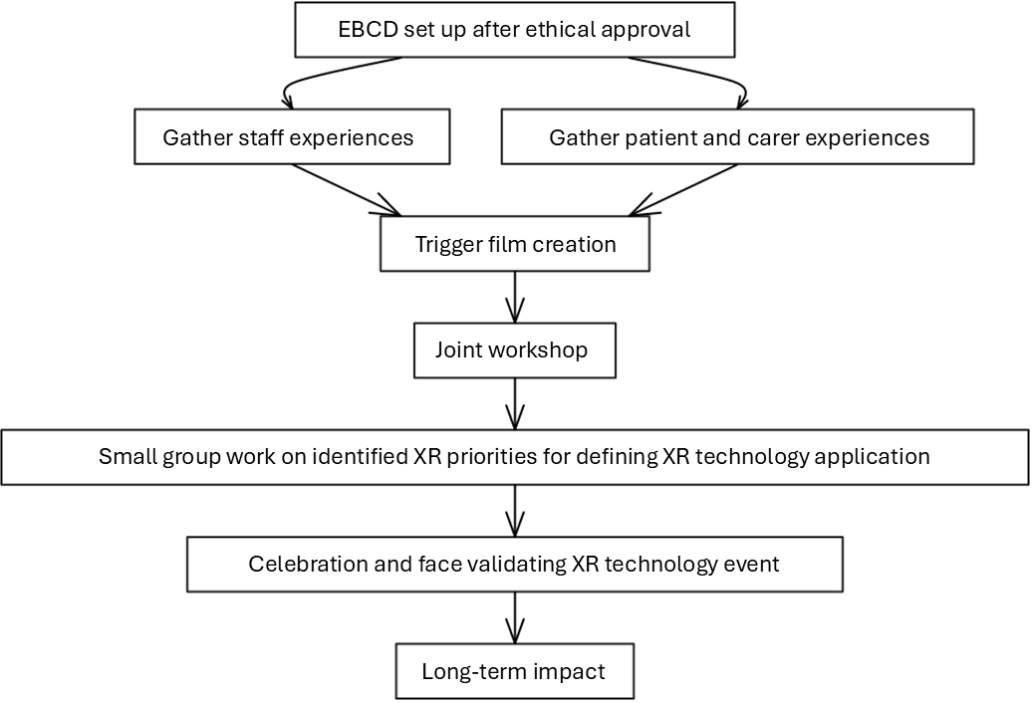
Flow diagram representing the experience-based co-design (EBCD) process. XR: extended reality.

This study aims to coproduce an at-home rehabilitation solution using XR biofeedback training under operant conditioning for functional limb weakness, integrated into the FND stepped care framework [86] for multidisciplinary treatment and rehabilitation. The goal also ensures that at-home rehabilitation solutions are not only precise and effective but also sustainable, economically viable, and ethically sound. Our predesign study [35], funded by Engineering and Physical Sciences Research Council (EPSRC) Network+ funding, led to the development of this study, beginning with activity 1 (engagement with and preferences of people with lived experience and broader stakeholders). Activity 1 will focus on understanding and innovating at-home rehabilitation XR solutions for integration into the adult clinical pathway, starting with recruitment and consent once ethics approval is obtained. While *invention* refers to the act of creating something entirely original, *innovation* involves enhancing existing technologies in new ways to use them. Our innovation effort will ensure that the co-design is informed by people with lived experience and broader stakeholder engagement and preferences, leading to more effective and meaningful implementation. The EBCD process involves 3 phases (Figure 4): capture the experience, understand the experience, and improve the experience (with the final phase being addressed in activity 2). Activity 2 will translate at-home rehabilitation XR technology requirements from activity 1 into actionable items using the predesign framework [35], considering broader stakeholder input and health care economics. We anticipate diverse views on AI regulations and the cost-effectiveness of XR technologies in FND, with concerns about equity and economic viability, particularly in resource-limited settings [94]. Cost-effectiveness analyses (CEAs) often overlook ethical considerations, potentially perpetuating disparities, especially in conditions such as FND [95]. To address the predisposing, precipitating, and perpetuating factors in FND [96], we will integrate rehabilitation technologies into a disease severity–based stepped care framework for FND management [86]. This activity, focused on activity 2 (requirement translation of XR technologies in FMD), will deliver actionable items and prioritized steps to close the loop, incorporating equity weights for CEAs based on patient disease severity [97] within the stepped care framework [86].

### Activity 1, Phase 1 (Mo 1-12): Recruit Stakeholders and Capture the Experience

#### Overview

Building on the predesign framework from our EPSRC Network+ seed-funded project [35] (Multimedia Appendix 1), we aim to ensure that the XR platform is accessible, impactful, and tailored to patient needs. Figure 4 illustrates the EBCD process, adapted for FMD care with XR technology integration across the clinic-to-home continuum. This multistage approach, partly based on prior research work [98], focuses on improving the transition of FMD rehabilitation from clinic to home through collaboration among staff, patients, and caregivers. Each component of the EBCD process is described in detail in the following subsections.

#### Components of the EBCD Process

##### EBCD Set Up After Ethics Approval

In this initial phase, ethics approval is secured, and preparations begin to gather experiences from health care staff and patients with FMD and their carers.

##### Gather Staff Experiences

This stage involves in-depth interviews and observations with health care staff to identify emotional touchpoints and challenges in the FMD care process after diagnosis.

##### Gather Patient and Carer Experiences

The focus shifts to patients with FMD and their caregivers, who share their experiences through 12 to 15 filmed narrative interviews. These narratives capture their journey and key emotional moments after the FMD diagnosis.

##### Trigger Film Creation

A 30-minute “trigger film” is edited from the narrative interviews, allowing staff to connect more deeply with the experience of patients with FMD by seeing and hearing their perspectives directly.

##### Joint Workshop

This workshop brings together staff, patients with FMD, and carers to review the film and share insights. It is a collaborative session essential for identifying specific constructs for applying XR technology in the FMD care process, spanning from clinic to home.

##### Small-Group Work on Identified XR Priorities for Defining XR Technology Application

Small groups are formed to address specific XR constructs, such as a virtual cognitive behavioral therapy coach, XR force feedback games, and XR position feedback games. They operate in 4-week Agile sprints to define and refine XR technology applications that align with the needs identified in the joint workshop.

##### Celebration and Face-Validating XR Technology Event

After specific prototype developments and design verification, a face-validation and celebration event takes place. This event celebrates progress and fosters a sense of achievement among participants.

##### Long-Term Impact

While each 4-week sprint provides incremental improvements, the co-design process over a maximum of 3 years, culminating in construct validation, is expected to lead to a patient-centered XR technology platform tailored to enhance FMD care from clinic to home settings for clinical usability and ecological validity testing.

#### Addressing Accessibility, Inclusivity, and Equity

The XR platform will include personalization and accessibility features, such as adjustable haptic controls, audio guidance, and customizable displays, tailored to patients’ needs as identified in the EBCD process. We aim for a diverse participant base, ensuring that the technology is suitable for patients across various ages, cultural backgrounds, and socioeconomic statuses. By actively recruiting underrepresented groups, especially female individuals, in whom FND is more prevalent [99], through patient advocates and charities, we prioritize inclusivity in development. Continuous feedback for prototyping and design verification, with parallel face validation, will allow iterative refinement based on user requirements.

#### Partnership and Stakeholder Involvement Strategy

Industry partners Nudge Reality Ltd and HumanRobotiX Ltd as well as robotic rehabilitation and haptic technology experts will enhance the platform with design-for-manufacture principles. Collaboration with FND charities and clinical networks will ensure that patient perspectives remain central. Patient advocates Katerina Hatjipanagioti and Matthew Newsham, from the EPSRC Network+ seed-funded project [35], will support outreach and participant recruitment.

#### Ensuring Responsible Innovation

The EBCD process will ensure transparency, inclusivity, and accountability by documenting development and integrating feedback, resulting in an accessible, ethical XR platform for FMD rehabilitation from clinic to home settings, aimed at enhancing both quality of life and health equity.

### Activity 2, Phase 2 (Mo 1-48): Requirement Translation of XR Technologies in FMD

#### Overview

**Figure 5:**
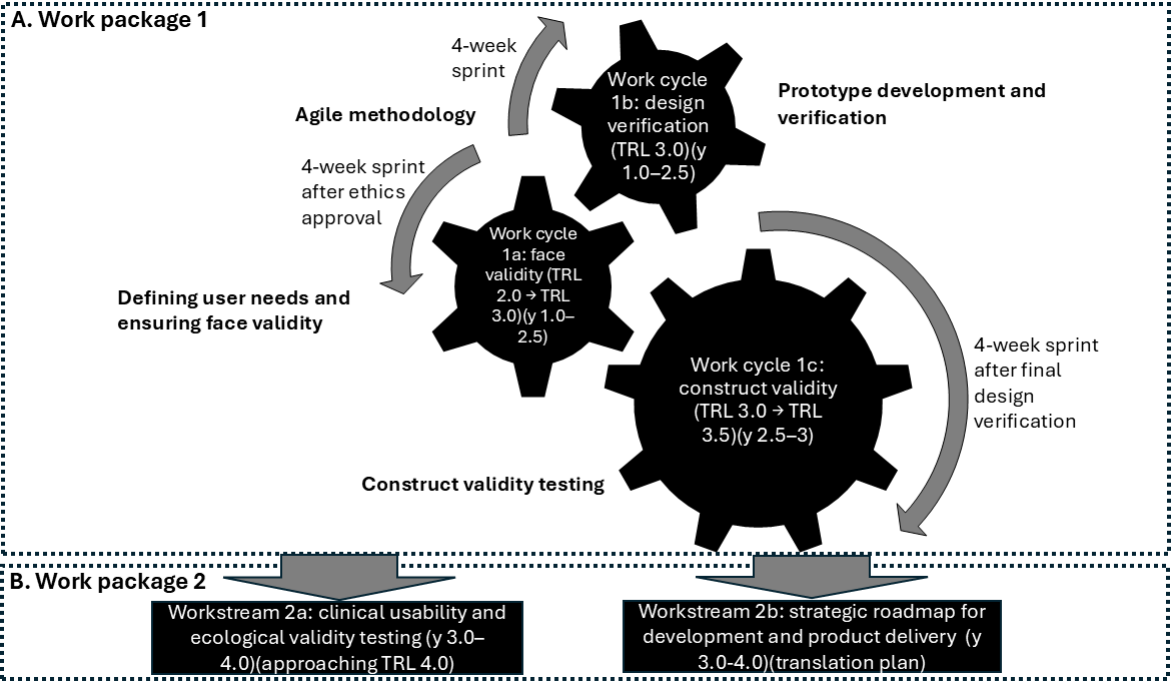
Overview of the work packages highlighting their structure, interactions, and key objectives within the project. TRL: technology readiness level.

To develop the XR platform for FMD rehabilitation from technology readiness level (TRL; a scale [ranging from 1 to 9] used to assess the maturity of a technology from concept [TRL 1] to full deployment [TRL 9]) 2.0 [35] to 3.5, we adopt a work cycle approach in work package 1, focusing on meeting user and stakeholder needs for translation and impact. Using coproduction, we ensure patient-centered quality by design (ISO 16355-1:2021) through patient and public involvement and engagement, iterative critical function validation (4-wk sprints), and testing (ISO 9241-220:2019) in an engineering laboratory with healthy participants. NVIDIA provides guardrails for physical AI through its Isaac platform and Jetson hardware, ensuring safe, reliable, and tested deployment of the XR platform. The work packages are illustrated in Figure 5 and are detailed in the Work Packages subsection, showing how quality function deployment (QFD; a structured method for translating user needs into engineering specifications during system development) will be operationalized alongside EBCD and 4-week Agile sprints during XR platform development.

#### EBCD Workshops: Capture of User Needs

EBCD workshops with patients, caregivers, and clinical staff will generate narrative data and structured feedback identifying key emotional touchpoints and rehabilitation needs. These are synthesized into user stories and functional requirements using thematic analysis in NVivo (Lumivero).

#### QFD Application: Translation Into Engineering Specifications

These user requirements are then mapped into a QFD matrix (house of quality) to prioritize technical features based on the frequency and criticality of patient and staff feedback. Relationships between user needs (eg, “easy-to-use interface” and “low visual discomfort”) and design components (eg, haptic resistance levels and XR visual rendering) are identified. Qualitative insights into quantifiable system parameters and performance metrics (eg, range of motion thresholds and feedback latency <100 ms) are translated. Each QFD matrix is reviewed by the cross-functional team (engineers, clinicians, and patient advocates) at the start of every sprint cycle, ensuring alignment between stakeholder input and design focus.

#### Agile Sprints: Implementation and Feedback Loops

Each 4-week Agile sprint consists of the following components:

- Week 1: sprint planning meeting, informed by QFD-derived priorities and usability issues identified in prior sprint testing
- Weeks 2 and 3: iterative prototype refinement, integrating hardware (HRX-1, brain imaging, and neurostimulation if necessary) and software (XR environment)
- Week 4: sprint review and testing session with 3 to 5 participants (patients or controls), using the System Usability Scale (SUS; a standardized 10-item questionnaire used to evaluate the usability of a product or system) and open-ended feedback

#### Validation and Loop Closure

Usability feedback is again categorized thematically and translated back into the next iteration’s QFD matrix, closing the loop between user experience and technical refinement. This process ensures that the prototype evolves in a traceable, patient-centered, and evidence-driven manner.

#### Work Packages

##### Work Package 1: Advancing Prototype and Construct Validity Through TRL 2.0 to 3.5

###### Work Cycle 1a: Defining User Needs and Ensuring Face Validity

This cycle engages patients with FMD, carers, health care staff, and FND charities through surveys, focus groups, and workshops following the EBCD approach (Figure 4). Insights inform critical function analysis and QFD to align XR technology with stakeholder needs, achieving face validity through iterative 4-week sprints.

###### Work Cycle 1b: Prototype Development and Verification

**Figure 6:**
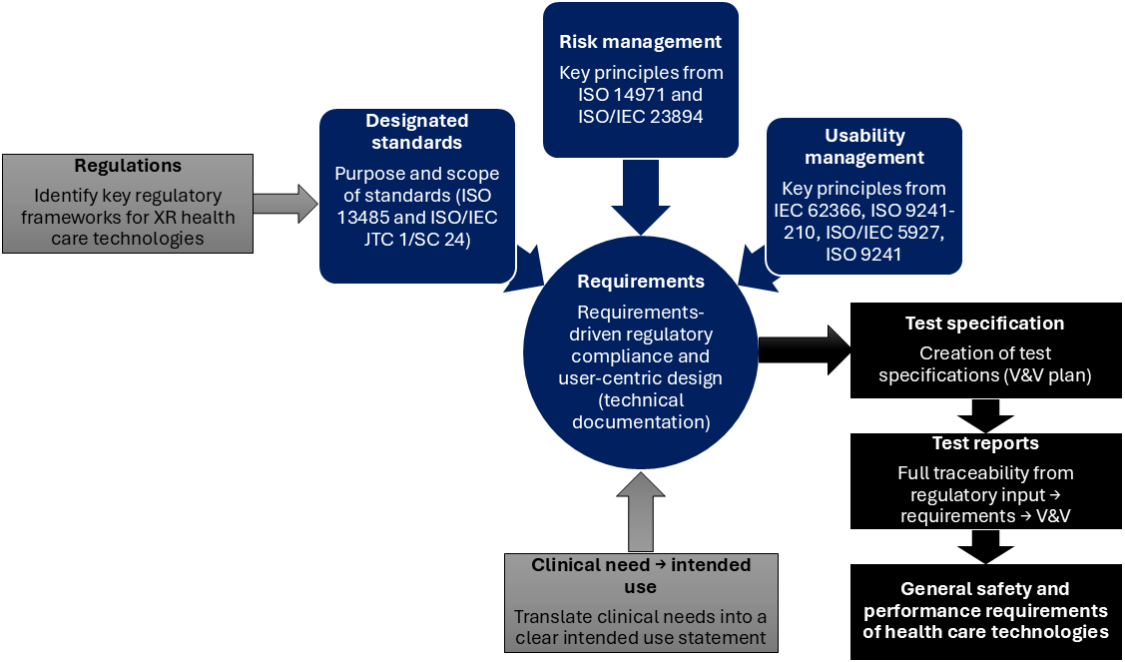
Verification and validation (V&V) of the artificial intelligence– and machine learning–enabled extended reality platform. System-level verification will be conducted to identify design flaws and resolve requirements. ISO/IEC 5927:2024 provides safety and well-being guidelines for immersive technologies such as virtual reality and augmented reality, covering risks such as motion sickness, eyestrain, and safe setup practices. The ISO 9241 series focus on ergonomics and usability in human-system interaction, with portions such as 9241-920 and 9241-940 specifically addressing the design and evaluation of haptic and tactile feedback. Together, these standards support the development of user-friendly, safe, and effective extended reality (XR) systems. IEC: International Electrotechnical Commission; ISO: International Organization for Standardization.

The HRX-1 device is reengineered and integrated with the Meta Quest 3 headset and wearable brain imaging capabilities, guided by QFD. A prototype characteristics matrix informs iterative development and verification through 4-week sprints to ensure compliance with design requirements (Figure 6). Expectation maximization (EM) is used in conjunction with the Kalman filter [3] when model parameters (eg, transition probabilities and noise covariances) are unknown and must be estimated from data. While the Kalman filter excels at real-time state estimation, assuming fixed parameters, EM is ideal for parameter estimation in probabilistic models, especially when working with incomplete or noisy data, such as neural signals. In brain state transitions (Figure 1), EM is used in switching state-space models to learn hidden brain state dynamics (eg, attention states) from fNIRS and EEG recordings, while the Kalman filter performs the real-time estimation of latent states once the model is trained [3]. These methods will be combined with EM performing offline learning, with the Kalman filter embedded in its expectation step for state inference [3]. This approach is well documented in neuroscience and signal processing literature [100,101].

For personalization, the XR platform will incorporate haptic device control algorithms that adapt both the difficulty of training tasks and the parameters of haptic feedback in real time [3]. Specifically, data streams from haptic device sensors (eg, motion and force input) and neuroimaging modalities (eg, fNIRS and EEG) will be used to infer user brain-behavior engagement, motor effort, and cognitive workload. A reinforcement learning framework will then adjust task parameters such as target accuracy thresholds, movement speed, or visual feedback gain based on performance trends and error profiles; for example, if a user demonstrates consistent overshooting or hesitation during reaching tasks, the system will gradually adjust the virtual target zones and reduce haptic resistance to facilitate motor learning while maintaining an appropriate level of challenge. Similarly, attention-aware modeling using switching state-space models will classify brain states associated with fatigue or disengagement, prompting breaks or switching to lower-load tasks. These adaptive control strategies will be trained offline using EM to learn individual response patterns and updated in deployment using embedded Kalman filtering for real-time state estimation [3]. This integration of AI and ML enables dynamic personalization of the rehabilitation experience, ensuring that the XR environment responds to each patient’s evolving abilities, optimizing both usability and therapeutic efficacy. At this stage, design-time guardrails must be established through trustworthy development pipelines that integrate third-party safety assessments (eg, MATLAB, Simulink, System Composer, and Requirements Toolbox) and follow robust processes focused on reliability and security in the AI- and ML-enabled model-based systems engineering of the XR platform.

###### Work Cycle 1c: Construct Validity Testing

SUS assessments and open-ended interviews will be conducted with healthy controls to refine sensor, effector, and AI and ML usability (Figure 6). Validation-time guardrails are enforced through hardware-in-the-loop high-fidelity simulation environments (eg, MATLAB, Simulink, System Composer, and Requirements Toolbox), enabling the testing of the AI- and ML-enabled XR platform across many real-world scenarios (eg, sensor fault [102], security, and authenticity [103]). Pilot studies will then assess convergent and divergent validity of brain responses (eg, beta oscillations and SMA efferent network activity from dFC) using the XR platform in an engineering laboratory environment.

##### Work Package 2: Advancing Usability, Validity, and Strategic Delivery

###### Workstream 2a: Clinical Usability and Ecological Validity Testing

Usability studies will be conducted with 50 patients with FMD for 3 alternate-day clinic visits, evaluating SUS scores and the platform’s clinical validity in a safe, controlled setting. Deployment-time guardrails will be responsible for executing real-time performance monitoring and error detection, as well as initiating fallback procedures during the XR platform operation under controlled clinical conditions.

###### Workstream 2b: Strategic Road Map for Development and Product Delivery

A translation and impact plan using the results from workstream 2a will be developed for future validation in a stepped care clinic-to-home framework (TRL ≥4.0). Guardrails for surveillance will ensure continuous monitoring, issue detection, regulatory compliance, and safe updates to maintain safety and performance. To support the scalability and real-world adoption of XR-based rehabilitation for functional limb weakness in FMD, this study will conduct a rigorous economic evaluation using the generalized risk-adjusted cost-effectiveness (GRACE; a method to adjust willingness-to-pay [WTP] thresholds by considering risk, severity, and outcome distributions) framework [97]. This approach improves upon traditional CEA by integrating patient-level risk preferences and variability in health outcomes, which are especially important in heterogeneous conditions such as FMD. A discrete choice experiment will be conducted with 50 patients diagnosed with functional limb weakness to estimate their WTP thresholds for home-based XR therapy and to quantify the quality-of-life impact of untreated symptoms. We will quantify the perceived impact of untreated FMD symptoms (functional limb weakness) on quality of life; estimate patients’ WTP thresholds for home-based XR rehabilitation; and identify trade-offs that patients are willing to make between convenience, effectiveness, risk, and cost. This will allow us to elicit patient-centered utility values and incorporate preference heterogeneity into the economic model. The results will inform a patient-centered utility model that accounts for preference heterogeneity. Unlike traditional CEA that relies on average quality-adjusted life years (QALYs), the GRACE framework computes generalized risk-adjusted QALYs, incorporating outcome variance (uncertainty in outcomes), skewness (asymmetric benefits), and kurtosis (extreme outcome tails) to better reflect patient-specific risk profiles and treatment uncertainty. This allows us to reflect not only the mean effectiveness of the XR intervention but also its distributional effects, including benefits for those at higher risk or with more severe impairment. This enables the calculation of a risk-adjusted WTP threshold (*K_GRACE_*) using the following formula:

*K_GRACE_* ≥ *ΔCost* / *Δ*(*GRAQALYs*)

where *ΔCost* is the incremental cost of the XR intervention compared to standard care, *GRAQALYs* stands for generalized risk-adjusted (for baseline severity, disability, and risk preferences) QALYs, and *Δ*(*GRAQALYs*) is the gain in risk-adjusted health benefits (QALYs). By integrating clinical outcomes, cost, and patient risk tolerance, this approach provides a robust basis for determining value for money, guiding pricing, reimbursement, and the equitable implementation of XR rehabilitation in FND care pathways. Ultimately, this integrated approach ensures that home-based XR rehabilitation is not only clinically promising but also economically justifiable and aligned with real-world patient values.

##### Work Package 3: Team Management - Workstream 3: Maintain Strong Team Coordination

We will use weekly meetings, 4-week sprints, and cross-team synchronization meetings held every 2 weeks to align development and clinical goals. Centralized tools, shared repositories, and cross-team workshops will enable transparent communication and knowledge sharing for risk management. Progress tracking through key performance indicators and Agile feedback loops will ensure seamless integration, address stakeholder feedback, and align priorities for timely XR platform delivery.

### Participants: Number and Diversity

#### Patients With FMD

We plan to recruit up to 50 patients with FMD to account for variability in symptoms such as functional weakness and dystonia, enabling subgroup analyses. This sample size is based on statistical power analysis using SUS scores from pilot testing across 3 XR tasks (hypnotic relaxation, position feedback, and force feedback) [35]. An effect size (Cohen *d*) of 0.81 was calculated, indicating a large difference between usability scores for position feedback and force feedback tasks. With a significance level of .05 and 80% power, approximately 15 participants per group are needed for usability testing. Recruiting 50 patients ensures that the study is adequately powered to detect meaningful differences and supports robust platform refinement and clinical translation.

#### Healthy Participants

Approximately 15 healthy participants will be included in the pilot studies in work package 1 to validate usability, construct validity, and core platform functions through a test-retest study over 6 repeats. Participants will be drawn from diverse backgrounds, focusing on inclusivity across socioeconomic groups, ages, and underrepresented populations. Given the higher prevalence of FMD in female individuals, recruitment efforts will emphasize female participation.

### Recruitment and Engagement

Collaboration with FND charities (eg, FND North and FND Action) and patient advocates will support recruitment, ensuring diverse participation through purposive sampling. EBCD processes will be used to coproduce the XR platform with participants, aligning the development with stakeholder needs.

### Procedures

Initial studies will take place in controlled laboratory settings at the Lancashire Clinical Research Facility to prioritize safety. Studies involving patients with FMD will be conducted under clinical supervision.

### Areas of Substantial or Moderate Impact

Minimal physical risks are anticipated with XR use. Co-design efforts will address known discomforts such as eyestrain, and iterative feedback will ensure participant safety and well-being during XR tasks. Recognizing the vulnerability of patients with FMD, therapeutic environments within the XR platform will be carefully co-designed to prevent distress, guided by feedback from the EBCD process. Transparent consent processes, stringent deidentification protocols, and active stakeholder collaboration will mitigate ethical risks associated with the research.

## Results

**Predesign Study**

The predesign study, supported by EPSRC Network+ funding, investigated the usability of an XR neurotechnology platform for FND rehabilitation, focusing on biofeedback training for functional limb weakness [35] (Multimedia Appendix 1). In the first round of a Delphi survey (a structured expert consensus method involving multiple rounds of questionnaires to refine opinions or priorities) involving 20 participants with lived experience of FND, there was strong consensus on the potential of VR with haptics for biofeedback-based motor rehabilitation. Participants highlighted the immersive experience, real-time feedback, and customizable environments as key advantages for improving motor control in functional limb weakness. However, several barriers to adoption were identified including high equipment costs, limited accessibility through public health care systems, concerns around usability (eg, motion sickness, technical learning curve), and sensory overstimulation. These findings informed subsequent predesign study of a home-based rehabilitation system integrating operant conditioning within mixed-reality environments. Limited accessibility through public health care systems prompted the need for the integration of patient-centered cost-effectiveness analysis in coproduction that accounts for disease severity, disability, and individual preferences thereby addressing limitations in traditional QALY models.

The predesign study evaluated an XR-based biofeedback platform for upper limb rehabilitation in individuals with FND, developed through co-design with patients and clinicians. Among the 3 tested XR tasks, high usability scores (>85) were recorded for XR position feedback task, indicating excellent user satisfaction. By contrast, the VR relaxation task produced mixed reactions due to motion discomfort, and the XR force feedback task showed varied results depending on individual motor impairments, such as functional dystonia, emphasizing the need for personalized approaches. Qualitative feedback highlighted the importance of comfort, immersive content, personalization, and accessibility. Therefore, predesign study findings emphasized the potential of XR in FND rehabilitation but highlighted the need for personalized approaches to meet diverse patient needs in FND. The predesign study further demonstrated the promise of XR technology for functional motor disorder rehabilitation by embedding biofeedback training into a patient-centered game design framework. EBCD and QFD emerged as valuable methods for coproduction and to systematically address usability barriers. To deepen personalization, this study will incorporate portable brain imaging to assess dFC in key regions during XR intervention, enabling individually responsive dosing. Moreover, the themes for improvement identified from the predesign study included customization, comfort, accessibility, and technological quality.

### This Study

This coproduction study is projected to be completed within 4 years, with results anticipated for publication in 2029.

## Discussion

### Summary

The REACT2HOME proposal lays the groundwork for a personalized, home-based XR rehabilitation approach tailored to individuals with functional limb weakness [89]. This subtype of FMD has a clearly defined semiology and lends itself well to structured motor retraining in XR. Our approach leverages operant conditioning and dynamic feedback loops to recalibrate predictive motor models, targeting the neural circuits implicated in agency and sensory attenuation. While full-scale clinical efficacy remains to be demonstrated, early indicators from usability testing and prior XR and VR studies suggest that targeted digital biofeedback could serve as a promising adjunct to multidisciplinary FND treatment [35,74,104]. Given the variability in FMD presentation, we expect recovery trajectories to be most significant in patients with functional weakness who retain volitional motor capacity but show impairment in motor planning, agency, or learned nonuse patterns [90].

The predesign study demonstrated the potential of XR technology as a transformative tool for FND rehabilitation [35]. The perception-action cycle, which is central to human adaptation, is disrupted in FND due to maladaptive learning processes that diminish agency and disrupt motor control. By integrating biofeedback training with immersive XR environments, this approach addresses both motor and sensory dysfunctions, presenting a novel therapeutic avenue. The findings align with the results of our Delphi survey (a structured expert consensus method involving multiple rounds of questionnaires to refine opinions or priorities) on VR haptic biofeedback [105], which highlighted the relevance of XR technology in improving motor control for patients with FMD. Participants in both the predesign and Delphi studies emphasized the immersive and customizable nature of XR environments as a critical factor for engagement and recovery. However, barriers such as cost, accessibility, and motion sickness need to be addressed to enable widespread adoption, as reflected in both studies.

The usability testing revealed higher satisfaction with VR relaxation and XR position feedback tasks, aligning with the Delphi findings that underscored the importance of comfort and accessibility [35]. However, the variability in XR force feedback usability, particularly among participants with conditions such as functional dystonia, emphasizes the need for personalized approaches. These results support the Delphi survey recommendation for task-specific customizations and accessibility enhancements to ensure broader usability across diverse patient needs. The predesign study also underscored the importance of coproduction in designing rehabilitation technologies, as demonstrated by the mixed methods approach and Delphi framework. Engaging patients with FND and clinicians in the design process ensures that the technology addresses real-world challenges, such as motion sickness and sensory overload, highlighted in both the predesign and Delphi studies.

This study builds on prior predesign work in FND rehabilitation [35] and integrates key pathophysiological insights specific to functional limb weakness, namely deficits in sensory attenuation, motor intention encoding, and agency disruption, to refine the XR platform for this targeted clinical application. By aligning the XR interventions with these mechanistic targets, such as prediction error recalibration and intention-action coupling, the platform aims to deliver therapeutically relevant biofeedback experiences. Patient-specific adjustments, including calibration for individual motor impairments and optimization of sensory inputs, are incorporated to enhance comfort, usability, and therapeutic responsiveness. Using a structured EBCD process, our approach supports meaningful engagement with patients and caregivers, ensuring that the technology remains responsive to their evolving needs. The platform’s modular design, guided by insights from both iterative usability testing and Delphi surveys, positions it for broader integration into a stepped care FND model, facilitating sustained improvements in functional outcomes and patient experience from clinic to home.

While several VR interventions have been explored for FND [74], most existing tools are based on cognitive behavioral therapy principles, exposure-based paradigms, or distraction techniques aimed at symptom habituation or anxiety reduction; for example, VR protocols often focus on mirror visual feedback and exposure therapy to reduce symptom expression during tasks [104]. By contrast, our XR platform introduces a mechanistically grounded operant conditioning framework designed to recalibrate sensorimotor prediction errors and restore the sense of agency. This is achieved through the real-time, implicit reinforcement of volitional motor behaviors using personalized visual and haptic feedback. Rather than focusing solely on cognitive reframing, the platform targets disrupted forward models and sensory attenuation mechanisms, engaging dynamic brain-behavior loops through closed-loop feedback for implicit learning [3]. In addition, our use of wearable neuroimaging and dFC analysis to guide task adaptation and monitor agency-related brain networks (eg, SMA-rTPJ connectivity) represents a significant technical and conceptual advancement over prior VR applications in FND rehabilitation. This biologically informed, adaptive XR approach fills a critical gap between top-down cognitive interventions and bottom-up motor retraining strategies. However, we recognize that FMD encompasses a broad spectrum of presentations, including functional tremors and dystonia, which may respond differently to XR-based interventions. Therefore, while the current design is optimized for patients with functional limb weakness, future iterations of the platform could include additional modules tailored to other FMD subtypes; for example, tremor-specific VR scenarios could be introduced using wearable sensors and visual feedback techniques.

As AI- and ML-enabled XR technologies continue to evolve, their adaptability offers significant promise for addressing the heterogeneity of FMD, for example, by using physical AI to address real-time force feedback in functional dystonia [35]. Advances in wearable brain imaging, AI- and ML-enabled personalization, and modular haptic feedback design will facilitate expansion beyond single subtypes. By grounding these tools in robust clinical frameworks and coproduction with patients, AI- and ML-enabled XR platforms can support both therapeutic engagement and mechanistic understanding of symptom recovery in FND. In this context, our long-term vision is to incorporate this technology within a stepped care model [86], enabling equitable, scalable, and home-based rehabilitation for a broader population with FMD. Ongoing stakeholder engagement and iterative validation will be key to achieving this translational pathway. To ensure long-term sustainability beyond the 4-year study timeline, we are developing an implementation road map that aligns with the priorities of the NHS long-term plan for digital health and community-based care. Upon the successful demonstration of clinical efficacy and usability, we aim to transition the XR platform into validated digital therapeutics that can be adopted within existing care pathways for FND. We plan to partner with integrated care systems, NHS Trusts, and neurorehabilitation clinics to pilot deployment within stepped care service models. In parallel, we will engage early with commissioners, health economists, and digital health assessment frameworks (eg, the National Institute for Health and Care Excellence evidence standards framework for digital health technologies [106]) to ensure readiness for regulatory approval and health technology assessment. Reimbursement strategies will include positioning the platform under NHS tariff codes for remote rehabilitation and pursuing designation as an “innovation technology payment” candidate [107]. In addition, we will explore partnerships with insurers and digital health platforms to extend reach into community and private care settings. Open-source models for modular hardware components and software algorithms will also be evaluated to support broader dissemination and integration. These combined strategies will help embed this XR platform into routine FND care, making it a scalable and sustainable solution beyond the initial funding period.

### Conclusions

The REACT2HOME study presents a novel, interdisciplinary approach to at-home neurorehabilitation for functional limb weakness. Grounded in predictive coding and agency-related neuroscience, the XR platform combines operant conditioning with adaptive feedback to restore volitional motor control and recalibrate disrupted sensorimotor processes. The methodology integrates EBCD, QFD, and Agile engineering sprints to ensure that the technology is user centered, technically robust, and clinically meaningful. Personalization is achieved through ML algorithms that dynamically adjust task difficulty and haptic feedback based on individual behavioral and neurophysiological responses. A rigorous risk management strategy aligned with ISO 14971 and ISO/IEC (International Electrotechnical Commission) 5927:2024 ensures safety and usability, while economic evaluation under the GRACE framework addresses long-term value, equity, and risk-adjusted cost-effectiveness. Importantly, the study establishes a road map for sustainable adoption through partnerships with health care providers, regulators, and payers, positioning XR-based rehabilitation as a scalable solution within stepped care models for FND. Through this protocol, we aim to transform how functional motor symptoms are managed, delivering evidence-based, accessible, and adaptive therapy directly into patients’ homes.

## Appendix

Multimedia Appendix 1 National Rehabilitation Centre Rehabilitation Technologies Conference 2024 presentation.

## Abbreviations

AI: artificial intelligence
CEA: cost-effectiveness analysis
dFC: dynamic functional connectivity
DMN: default mode network
DSM-5: Diagnostic and Statistical Manual of Mental Disorders (Fifth Edition)
EBCD: experience-based co-design
EEG: electroencephalography
EM: expectation maximization
EPSRC: Engineering and Physical Sciences Research Council
FLS: fundamentals of laparoscopic surgery
FMD: functional movement disorder
FND: functional neurological disorder
fNIRS: functional near-infrared spectroscopy
GRACE: generalized risk-adjusted cost-effectiveness
HMI: human-machine interface
ISO: International Organization for Standardization
M1: primary motor cortex
ML: machine learning
MRCP: movement-related cortical potential
NHS: National Health Service
PFC: prefrontal cortex
QALY: quality-adjusted life year
QFD: quality function deployment
RDoC: research domain criteria
rTPJ: right temporoparietal junction
SCAN: somato-cognitive action network
SMA: supplementary motor area
SUS: System Usability Scale
TBS: theta burst stimulation
TPJ: temporoparietal junction
TRL: technology readiness level
VR: virtual reality
WTP: willingness-to-pay
XR: extended reality

## Data Availability

The datasets generated or analyzed during this study are available in the GitHub repository [109].

## References

[1] Hallett M, Aybek S, Dworetzky BA, McWhirter L, Staab JP, Stone J (2022). Functional neurological disorder: new subtypes and shared mechanisms. Lancet Neurol.

[2] Edwards MJ, Adams RA, Brown H, Pareés I, Friston KJ (2012). A Bayesian account of 'hysteria'. Brain.

[3] Dutta A (2025). Neurocomputational mechanisms of sense of agency: literature review for integrating predictive coding and adaptive control in human-machine interfaces. Brain Sci.

[4] Perez DL (2020). Persistence of the 'broken escalator' phenomenon in functional gait disorder: mechanistic insights. Brain.

[5] Gilmour GS, Lidstone SC, Lang AE The diagnosis of functional movement disorder. Practical Neurology.

[6] Pringsheim T, Edwards M (2017). Functional movement disorders: five new things. Neurol Clin Pract.

[7] Wolpert DM, Ghahramani Z (2000). Computational principles of movement neuroscience. Nat Neurosci.

[8] Pareés I, Brown H, Nuruki A, Adams RA, Davare M, Bhatia KP, Friston K, Edwards MJ (2014). Loss of sensory attenuation in patients with functional (psychogenic) movement disorders. Brain.

[9] Brown H, Adams RA, Parees I, Edwards M, Friston K (2013). Active inference, sensory attenuation and illusions. Cogn Process.

[10] Hughes G, Desantis A, Waszak F (2013). Mechanisms of intentional binding and sensory attenuation: the role of temporal prediction, temporal control, identity prediction, and motor prediction. Psychol Bull.

[11] McNaughton D, Hope R, Gray E, Xavier F, Beath A, Jones M (2023). Methodological considerations for the force-matching task. Behav Res Methods.

[12] Voss M, Bays PM, Rothwell JC, Wolpert DM (2007). An improvement in perception of self-generated tactile stimuli following theta-burst stimulation of primary motor cortex. Neuropsychologia.

[13] Blakemore SJ, Frith CD, Wolpert DM (2001). The cerebellum is involved in predicting the sensory consequences of action. Neuroreport.

[14] (2009). Exploring the Thalamus and Its Role in Cortical Function. 2nd edition.

[15] Hua L, Adams RA, Grent-'t-Jong T, Gajwani R, Gross J, Gumley A, Krishnadas R, Lawrie S, Schultze-Lutter F, Schwannauer M, Uhlhaas P (2023). Thalamo-cortical circuits during sensory attenuation in emerging psychosis: a combined magnetoencephalography and dynamic causal modelling study. Schizophrenia (Heidelb).

[16] Walia P, Ghosh A, Singh S, Dutta A (2022). Portable neuroimaging-guided noninvasive brain stimulation of the Cortico-Cerebello-Thalamo-Cortical Loop-Hypothesis and theory in cannabis use disorder. Brain Sci.

[17] Maurer CW, LaFaver K, Ameli R, Epstein SA, Hallett M, Horovitz SG (2016). Impaired self-agency in functional movement disorders: a resting-state fMRI study. Neurology.

[18] Tan S, Jia Y, Jariwala N, Zhang Z, Brent K, Houde J, Nagarajan S, Subramaniam K (2024). A randomised controlled trial investigating the causal role of the medial prefrontal cortex in mediating self-agency during speech monitoring and reality monitoring. Sci Rep.

[19] Moore JW (2016). What is the sense of agency and why does it matter?. Front Psychol.

[20] Bühler J, Weber S, Loukas S, Walther S, Aybek S (2024). Non-invasive neuromodulation of the right temporoparietal junction using theta-burst stimulation in functional neurological disorder. BMJ Neurol Open.

[21] Zito GA, Wiest R, Aybek S (2020). Neural correlates of sense of agency in motor control: a neuroimaging meta-analysis. PLoS One.

[22] Dutta A (2025). Bayesian predictive coding hypothesis: brain as observer’s key role in insight. Med Hypotheses.

[23] Kühn S, Brass M, Haggard P (2013). Feeling in control: neural correlates of experience of agency. Cortex.

[24] Dutta A (2025). 'Hyperbinding' in functional movement disorders: role of supplementary motor area efferent signalling. Brain Commun.

[25] Hillyard SA, Vogel EK, Luck SJ (1998). Sensory gain control (amplification) as a mechanism of selective attention: electrophysiological and neuroimaging evidence. Philos Trans R Soc Lond B Biol Sci.

[26] Maier ME, Yeung N, Steinhauser M (2011). Error-related brain activity and adjustments of selective attention following errors. Neuroimage.

[27] Berger CC, Ehrsson HH (2013). Mental imagery changes multisensory perception. Curr Biol.

[28] Strube A, Rose M, Fazeli S, Büchel C (2021). The temporal and spectral characteristics of expectations and prediction errors in pain and thermoception. Elife.

[29] Steinruecke M, Mason I, Keen M, McWhirter L, Carson AJ, Stone J, Hoeritzauer I (2024). Pain and functional neurological disorder: a systematic review and meta-analysis. J Neurol Neurosurg Psychiatry.

[30] Dutta A, Lahiri U, Das A, Nitsche MA, Guiraud D (2014). Post-stroke balance rehabilitation under multi-level electrotherapy: a conceptual review. Front Neurosci.

[31] Kumar D, Sinha N, Dutta A, Lahiri U (2019). Virtual reality-based balance training system augmented with operant conditioning paradigm. Biomed Eng Online.

[32] Turner JA, Chapman RC (1982). Psychological interventions for chronic pain: a critical review. II. Operant conditioning, hypnosis, and cognitive-behavioral therapy. Pain.

[33] Walia P, Fu Y, Norfleet J, Schwaitzberg SD, Intes X, De S, Cavuoto L, Dutta A (2022). Error-related brain state analysis using electroencephalography in conjunction with functional near-infrared spectroscopy during a complex surgical motor task. Brain Inform.

[34] Dutta A, Boulenouar RS, Guiraud D, Nitsche MA (2014). Delineating the effects of anodal transcranial direct current stimulation on myoelectric control based on slow cortical potentials. Annu Int Conf IEEE Eng Med Biol Soc.

[35] Dutta A, Hatjipanagioti K, Newsham MA, Leyland L, Rickson L, Buchanan A, Farkhatdinov I, Twamley J, Das A (2025). Extended reality biofeedback for functional upper limb weakness: mixed methods usability evaluation. JMIR XR Spatial Comput.

[36] Gordon EM, Chauvin RJ, Van AN, Rajesh A, Nielsen A, Newbold DJ, Lynch CJ, Seider NA, Krimmel SR, Scheidter KM, Monk J, Miller RL, Metoki A, Montez DF, Zheng A, Elbau I, Madison T, Nishino T, Myers MJ, Kaplan S, Badke D'Andrea C, Demeter DV, Feigelis M, Ramirez JS, Xu T, Barch DM, Smyser CD, Rogers CE, Zimmermann J, Botteron KN, Pruett JR, Willie JT, Brunner P, Shimony JS, Kay BP, Marek S, Norris SA, Gratton C, Sylvester CM, Power JD, Liston C, Greene DJ, Roland JL, Petersen SE, Raichle ME, Laumann TO, Fair DA, Dosenbach NU (2023). A somato-cognitive action network alternates with effector regions in motor cortex. Nature.

[37] Pastötter B, Weissbach A, Takacs A, Moyé J, Verrel J, Chwolka F, Friedrich J, Paulus T, Zittel S, Bäumer T, Frings C, Beste C, Münchau A (2024). Increased beta synchronization underlies perception-action hyperbinding in functional movement disorders. Brain Commun.

[38] Di Vico IA, Cirillo G, Tessitore A, Siciliano M, Venturelli M, Falup-Pecurariu C, Tedeschi G, Morgante F, Tinazzi M (2021). Fatigue in hypokinetic, hyperkinetic, and functional movement disorders. Parkinsonism Relat Disord.

[39] Kamat A, Makled B, Norfleet J, Schwaitzberg SD, Intes X, De S, Dutta A (2022). Directed information flow during laparoscopic surgical skill acquisition dissociated skill level and medical simulation technology. NPJ Sci Learn.

[40] Kamat A, Rahul R, Dutta A, Cavuoto L, Kruger U, Burke H, Hackett M, Norfleet J, Schwaitzberg S, De S Dynamic directed functional connectivity as a neural biomarker for objective motor skill assessment. arXiv.

[41] Seghezzi S, Zapparoli L (2020). Predicting the sensory consequences of self-generated actions: pre-supplementary motor area as supra-modal hub in the sense of agency experience. Brain Sci.

[42] Haggard P (2008). Human volition: towards a neuroscience of will. Nat Rev Neurosci.

[43] Haggard P, Whitford B (2004). Supplementary motor area provides an efferent signal for sensory suppression. Brain Res Cogn Brain Res.

[44] Teodoro T, Meppelink AM, Little S, Grant R, Nielsen G, Macerollo A, Pareés I, Edwards MJ (2018). Abnormal beta power is a hallmark of explicit movement control in functional movement disorders. Neurology.

[45] Lundqvist M, Miller EK, Nordmark J, Liljefors J, Herman P (2024). Beta: bursts of cognition. Trends Cogn Sci.

[46] Yin S, Liu Y, Ding M (2016). Amplitude of sensorimotor Mu Rhythm is correlated with BOLD from multiple brain regions: a simultaneous EEG-fMRI study. Front Hum Neurosci.

[47] Zeng Y, Sauseng P, Alamia A (2024). Alpha traveling waves during working memory: disentangling bottom-up gating and top-down gain control. J Neurosci.

[48] McFarland DJ, Miner LA, Vaughan TM, Wolpaw JR (2000). Mu and beta rhythm topographies during motor imagery and actual movements. Brain Topogr.

[49] Wischnewski M, Haigh ZJ, Shirinpour S, Alekseichuk I, Opitz A (2022). The phase of sensorimotor mu and beta oscillations has the opposite effect on corticospinal excitability. Brain Stimul.

[50] Zito GA, de Sousa Ribeiro R, Kamal E, Ledergerber D, Imbach L, Polania R (2023). Self-modulation of the sense of agency via neurofeedback enhances sensory-guided behavioral control. Cereb Cortex.

[51] Seeley WW, Menon V, Schatzberg AF, Keller J, Glover GH, Kenna H, Reiss AL, Greicius MD (2007). Dissociable intrinsic connectivity networks for salience processing and executive control. J Neurosci.

[52] Morris LS, To B, Baek K, Chang-Webb YC, Mitchell S, Strelchuk D, Mikheenko Y, Phillips W, Zandi M, Jenaway A, Walsh C, Voon V (2017). Disrupted avoidance learning in functional neurological disorder: implications for harm avoidance theories. Neuroimage Clin.

[53] Spagnolo PA, Parker J, Horovitz S, Hallett M (2021). Corticolimbic modulation via intermittent theta burst stimulation as a novel treatment for functional movement disorder: a proof-of-concept study. Brain Sci.

[54] Das A, Dutta A (2024). Functional seizure therapy via transauricular vagus nerve stimulation. Med Hypotheses.

[55] Skinner BF (1938). The Behavior Of Organisms An Experimental.

[56] Huvermann DM, Berlijn AM, Thieme A, Erdlenbruch F, Groiss SJ, Deistung A, Mittelstaedt M, Wondzinski E, Sievers H, Frank B, Göricke SL, Gliem M, Köhrmann M, Siebler M, Schnitzler A, Bellebaum C, Minnerop M, Timmann D, Peterburs J (2025). The cerebellum contributes to prediction error coding in reinforcement learning in humans. J Neurosci.

[57] Degras D, Ting CM, Ombao H (2022). Markov-switching state-space models with applications to neuroimaging. Comput Statist Data Anal.

[58] Nahab FB, Kundu P, Gallea C, Kakareka J, Pursley R, Pohida T, Miletta N, Friedman J, Hallett M (2011). The neural processes underlying self-agency. Cereb Cortex.

[59] Bréchet L, Brunet D, Birot G, Gruetter R, Michel CM, Jorge J (2019). Capturing the spatiotemporal dynamics of self-generated, task-initiated thoughts with EEG and fMRI. Neuroimage.

[60] L'Huillier JC, Jones CB, Fu Y, Myneni AA, De S, Cavuoto L, Dutta A, Stefanski M, Cooper CA, Schwaitzberg SD (2025). On the journey to measure cognitive expertise: what can functional imaging tell us?. Surgery.

[61] Ide JS, Li CR (2011). A cerebellar thalamic cortical circuit for error-related cognitive control. Neuroimage.

[62] Blakemore SJ, Oakley DA, Frith CD (2003). Delusions of alien control in the normal brain. Neuropsychologia.

[63] Batsikadze G, Rezaee Z, Chang DI, Gerwig M, Herlitze S, Dutta A, Nitsche MA, Timmann D (2019). Effects of cerebellar transcranial direct current stimulation on cerebellar-brain inhibition in humans: a systematic evaluation. Brain Stimul.

[64] Rezaee Z, Dutta A (2019). Cerebellar Lobules Optimal Stimulation (CLOS): a computational pipeline to optimize cerebellar lobule-specific electric field distribution. Front Neurosci.

[65] Sendhilnathan N, Semework M, Goldberg ME, Ipata AE (2020). Neural correlates of reinforcement learning in mid-lateral cerebellum. Neuron.

[66] Polito V, Barnier AJ, Woody EZ (2013). Developing the Sense of Agency Rating Scale (SOARS): an empirical measure of agency disruption in hypnosis. Conscious Cogn.

[67] Bréchet L, Ziegler DA, Simon AJ, Brunet D, Gazzaley A, Michel CM (2021). Reconfiguration of electroencephalography microstate networks after breath-focused, digital meditation training. Brain Connect.

[68] Panda R, Bharath RD, Upadhyay N, Mangalore S, Chennu S, Rao SL (2016). Temporal dynamics of the default mode network characterize meditation-induced alterations in consciousness. Front Hum Neurosci.

[69] Katayama H, Gianotti LR, Isotani T, Faber PL, Sasada K, Kinoshita T, Lehmann D (2007). Classes of multichannel EEG microstates in light and deep hypnotic conditions. Brain Topogr.

[70] Xu X, Yuan H, Lei X (2016). Activation and connectivity within the default mode network contribute independently to future-oriented thought. Sci Rep.

[71] Terhune DB, Cleeremans A, Raz A, Lynn SJ (2017). Hypnosis and top-down regulation of consciousness. Neurosci Biobehav Rev.

[72] Stone J, Sharpe M (2001). Hoover’s sign. Pract Neurol.

[73] Huys AC, Edwards MJ, Bhatia KP, Haggard P (2020). Modulation of reaction times and sense of agency via subliminal priming in functional movement disorders. Front Neurol.

[74] Brouwer D, Morrin H, Nicholson TR, Terhune DB, Schrijnemaekers M, Edwards MJ, Gelauff J, Shotbolt P (2024). Virtual reality in functional neurological disorder: a theoretical framework and research agenda for use in the real world. BMJ Neurol Open.

[75] Kranick SM, Moore JW, Yusuf N, Martinez VT, LaFaver K, Edwards MJ, Mehta AR, Collins P, Harrison NA, Haggard P, Hallett M, Voon V (2013). Action-effect binding is decreased in motor conversion disorder: implications for sense of agency. Mov Disord.

[76] Moore JW, Ruge D, Wenke D, Rothwell J, Haggard P (2010). Disrupting the experience of control in the human brain: pre-supplementary motor area contributes to the sense of agency. Proc Biol Sci.

[77] Pfurtscheller G, Neuper C, Brunner C, da Silva FL (2005). Beta rebound after different types of motor imagery in man. Neurosci Lett.

[78] Nakamura A, Suzuki Y, Milosevic M, Nomura T (2021). Long-lasting event-related beta synchronizations of electroencephalographic activity in response to support-surface perturbations during upright stance: a pilot study associating beta rebound and active monitoring in the intermittent postural control. Front Syst Neurosci.

[79] Jurkiewicz MT, Gaetz WC, Bostan AC, Cheyne D (2006). Post-movement beta rebound is generated in motor cortex: evidence from neuromagnetic recordings. Neuroimage.

[80] Moore JW, Obhi SS (2012). Intentional binding and the sense of agency: a review. Conscious Cogn.

[81] Moore JW, Middleton D, Haggard P, Fletcher PC (2012). Exploring implicit and explicit aspects of sense of agency. Conscious Cogn.

[82] Stone J, Edwards M (2012). Trick or treat? Showing patients with functional (psychogenic) motor symptoms their physical signs. Neurology.

[83] Macías-García D, Méndez-Del Barrio M, Canal-Rivero M, Muñoz-Delgado L, Adarmes-Gómez A, Jesús S, Ojeda-Lepe E, Carrillo-García F, Palomar FJ, Gómez-Campos FJ, Martin-Rodriguez JF, Crespo-Facorro B, Ruiz-Veguilla M, Mir P (2024). Combined physiotherapy and cognitive behavioral therapy for functional movement disorders: a randomized clinical trial. JAMA Neurol.

[84] Lord Darzi’s independent investigation into NHS performance. House of Commons, UK.

[85] PM launches new era for NHS with easier care in neighbourhoods. Government of UK.

[86] Taing M, Sztainert T, Harley M, Allen K, Moore F (2025). Applying a stepped-care framework for functional neurological disorder management. Can J Neurol Sci.

[87] O'Neal MA, Baslet GC, Polich GR, Raynor GS, Dworetzky BA (2021). Functional neurologic disorders: the need for a model of care. Neurol Clin Pract.

[88] Ackerley S, Wilson N, Boland P, Peel R, Connell L (2024). NeuroRehabilitation OnLine: description of a regional multidisciplinary group telerehabilitation innovation for stroke and neurological conditions using the template for intervention description and replication checklist. Digit Health.

[89] McWhirter L, Stone J, Sandercock P, Whiteley W (2011). Hoover's sign for the diagnosis of functional weakness: a prospective unblinded cohort study in patients with suspected stroke. J Psychosom Res.

[90] Sanyal R, Raseta M, Natarajan I, Roffe C (2022). The use of hypnotherapy as treatment for functional stroke: a case series from a single center in the UK. Int J Stroke.

[91] Research Domain Criteria (RDoC). National Institute of Mental Health.

[92] Spagnolo PA, Garvey M, Hallett M (2021). A dimensional approach to functional movement disorders: heresy or opportunity. Neurosci Biobehav Rev.

[93] EBCD: experience based co-design toolkit. Picker Institute.

[94] Optimal clinical pathway for adults with functional neurological disorder FND. National Neurosciences Advisory Group.

[95] Canna M, Seligman R (2020). Dealing with the unknown. Functional neurological disorder (FND) and the conversion of cultural meaning. Soc Sci Med.

[96] Merritt Millman L, Short E, Ward E, Sun Y, Stanton B, Bradley-Westguard A, Goldstein L, Winston J, Mehta M, Nicholson T, Reinders S, David A, Edwards M, Chalder T, Hotopf M, Pick S (2023). 13 Predisposing, precipitating and perpetuating factors in functional neurological disorder: a pilot study. J Neurol Neurosurg Psychiatry.

[97] Lakdawalla DN, Phelps CE (2022). A guide to extending and implementing generalized risk-adjusted cost-effectiveness (GRACE). Eur J Health Econ.

[98] Twamley J, Monks R, Beaver K (2023). Using experience-based co-design to prioritise areas for improvement for patients recovering from critical illness. Intensive Crit Care Nurs.

[99] McLoughlin C, Hoeritzauer I, Cabreira V, Aybek S, Adams C, Alty J, Ball HA, Baker J, Bullock K, Burness C, Dworetzky BA, Finkelstein S, Garcin B, Gelauff J, Goldstein LH, Jordbru A, Huys AM, Laffan A, Lidstone SC, Linden SC, Ludwig L, Maggio J, Morgante F, Mallam E, Nicholson C, O'Neal M, O'Sullivan S, Pareés I, Petrochilos P, Pick S, Phillips W, Roelofs K, Newby R, Stanton B, Gray C, Joyce EM, Tijssen MA, Chalder T, McCormick M, Gardiner P, Bègue I, Tuttle MC, Williams I, McRae S, Voon V, McWhirter L (2023). Functional neurological disorder is a feminist issue. J Neurol Neurosurg Psychiatry.

[100] Ghahramani Z, Hinton GE (2000). Variational learning for switching state-space models. Neural Comput.

[101] Ghahramani Z, Roweis ST (1998). Learning nonlinear dynamical systems using an EM algorithm. Proceedings of the 12th International Conference on Neural Information Processing Systems.

[102] Dutta A, Koerding K, Perreault E, Hargrove L (2011). Sensor-fault tolerant control of a powered lower limb prosthesis by mixing mode-specific adaptive Kalman filters. Annu Int Conf IEEE Eng Med Biol Soc.

[103] Dutta A (2020). Brain-computer interface spellers for communication: why we need to address their security and authenticity. Brain Sci.

[104] Bullock K, Won AS, Bailenson J, Friedman R (2020). Virtual reality-delivered mirror visual feedback and exposure therapy for FND: a midpoint report of a randomized controlled feasibility study. J Neuropsychiatry Clin Neurosci.

[105] Das A, Newsham M, Hatjipanagioti K, Buchanan A, Farkhatdinov I, Dutta A (2024). VR haptics biofeedback training for functional limb weakness: insights from the first round of a Delphi survey. Proceedings of the 2024 Conference on NRC Rehabilitation Technologies.

[106] Evidence standards framework (ESF) for digital health technologies. NICE.

[107] Innovation and technology payment. NHS England.

[108] Microsoft Copilot, your new study tool. University of Birmingham.

[109] Siagnos / ICRA2025. GitHub.

